# Dispersal of Adult Black Marlin (*Istiompax indica*) from a Great Barrier Reef Spawning Aggregation

**DOI:** 10.1371/journal.pone.0031629

**Published:** 2012-02-21

**Authors:** Michael L. Domeier, Peter Speare

**Affiliations:** 1 Marine Conservation Science Institute, Fallbrook, California, United States of America; 2 Australian Institute of Marine Science, Townsville, Queensland, Australia; Institute of Marine Research, Norway

## Abstract

The black marlin (*Istiompax indica*) is one of the largest bony fishes in the world with females capable of reaching a mass of over 700 kg. This highly migratory predator occurs in the tropical regions of the Pacific and Indian Oceans, and is the target of regional recreational and commercial fisheries. Through the sampling of ichthyoplankton and ovaries we provide evidence that the relatively high seasonal abundance of black marlin off the Great Barrier Reef is, in fact, a spawning aggregation. Furthermore, through the tracking of individual black marlin via satellite popup tags, we document the dispersal of adult black marlin away from the spawning aggregation, thereby identifying the catchment area for this spawning stock. Although tag shedding is an issue when studying billfish, we tentatively identify the catchment area for this stock of black marlin to extend throughout the Coral Sea, including the waters of Papua New Guinea, the Solomon Islands, Micronesia, New Caledonia, Kiribati, Vanuatu, Fiji, Tuvalu and Nauru.

## Introduction

Identifying spawning regions is an important step in defining the spatial distribution of separate stocks of the same fish species. For highly migratory fishes, different stocks can mingle within feeding regions but then separate and exhibit natal homing to widely separated spawning grounds (e.g. Atlantic bluefin tuna –[1], [2]). Although tagging studies are effective at identifying patterns of fish movement, complementary studies must be undertaken to determine the motivation for movement to specific regions. Sampling of gonads and larvae are necessary when attempting to discern spawning season, spawning sites and spawning stocks. Although spawning stocks may not be genetically distinct they are important units for fishery management.

Some fish species gather in unusual densities at very specific sites and seasons to spawn. This phenomenon, termed a spawning aggregation [3], is commonly exhibited in certain families of coral reef fish but is far less frequently identified in pelagic species. The fact that spawning aggregations are very predictable in time and space creates a situation where directed fisheries can rapidly deplete a stock to the point of extirpation [4]. Spawning aggregations also create a valuable research opportunity since a large sample can be efficiently and economically tagged to study the geographic extent of the spawning stock. An unusual gathering of conspecific individuals may not always be for the purpose of spawning so spawning must be confirmed via direct or indirect means. Three methods have been proposed to confirm a spawning aggregation: 1) observation of spawning, 2) observation of hydrated eggs and 3) observation of post-ovulatory follicles [5].

The black marlin (*Istiompax indica*) is one of the largest bony fishes in the world with females capable of reaching a mass of over 700 kg [6]. This species ranges throughout the tropical Pacific and Indian Oceans with seasonal density hotspots occurring in the East China Sea, the northwest Coral Sea, the Arafura Sea, the Sulu Sea, the Celebes Sea, near Taiwan, northwestern Australia and Panama [7]. The presence of gravid females, and in some cases larvae, have suggested that seasonal aggregations of black marlin off the Great Barrier Reef (GBR) (October-December – [6], [8], [9]), Taiwan (August-October – [6]) and Hainan Island (May-June – [10]–[12]) may be spawning aggregations. The seasonal presence of black marlin off the GBR was once the focus of an important commercial fishery [13] and continues to be an economically important recreational fishery.

Leis et al. [9] conducted ichthyoplankton tows in the vicinity of the GBR recreational fleet that targets black marlin. They identified a maximum abundance of istiophorid (billfish) larvae immediately seaward of the Ribbon Reefs compared to adjacent sampling sites further offshore and inside the GBR lagoon. They concluded that the billfish spawn, or at least the eggs hatch, immediately seaward of the barrier reefs. They collected three distinct species of billfish larvae, two of which were known (blue marlin and sailfish). Through the process of elimination they concluded that the 3rd species was black marlin although the species identification was never subsequently confirmed. The concurrent presence of black marlin adults and presumed black marlin larvae supports the suggestion that the high seasonal density of fish is a spawning aggregation.

Through an integrated study of black marlin reproductive biology, larval sampling and adult black marlin tagging, we present evidence of spawning and document the geographic range of the black marlin stock that frequents the GBR.

## Materials and Methods

### Gonad sampling

**Figure 1:**
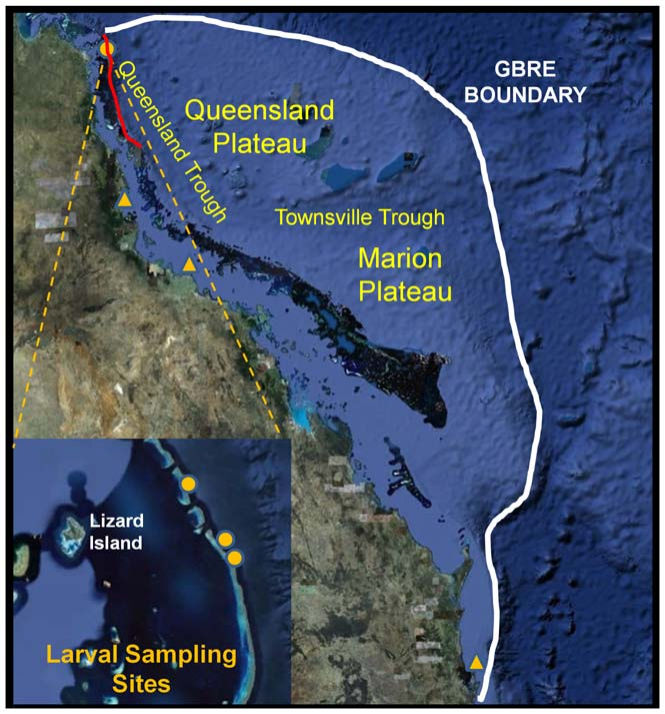
The white line outlines the Great Barrier Reef Environment (GBRE); a region of barrier reef and adjacent habitats considered important for black marlin spawning. Orange triangles are inshore gonad sampling sites (north to south: Dunk Island, Cape Bowling Green, Cape Moreton). Orange circles are larval sampling sites (inset north to south: Yonge Reef, First Corner, Second Corner). Red line is region of adult gonad sampling and PSAT tagging. Data SIO, NOAA, U.S. Navy, NGA, GEBCO; Image ©2011 TerraMetrics; Image © 2011 DigitalGlobe; © 2011 Cnes/Spot IMage.

Adult and juvenile fish were obtained between 1987 and 1991 from the recreational fishery operating along the Queensland coast, eastern Australia. Thirty males between 8.5 kg and 83.5 kg and 26 females (11.6–64 kg) were available from the nearshore fishing grounds off Cape Moreton, Dunk Island and Cape Bowling Green (Fig. 1). Twenty five large females (295–525 kg) and 3 males (59–158 kg) were secured from the Lizard Island Game Fish Club's annual October tournaments off the Ribbon reefs.

Date of capture, weight (kg), lower jaw-fork length (LJFL cm) and gonad weights (gms) were recorded for each specimen. Gonad tissue was routinely taken from the mid-section of a gonad, randomly left or right, and placed directly into 10% neutral buffered formalin solution. Subsequent histological preparation employed staged alcohol dehydration and wax embedded tissue sectioned at 5 µm prior to staining with haematoxylin and counter-staining with eosin.

A software driven computer dissecting microscope was utilized for processing the histological sections to recover imagery, oocyte counts and measurements. Three areas from each histological slide preparation were randomly selected at 40X magnification and imaged for later counting of the observed development stages (after [14]). Oocyte counts were made across each image and measurements (diameter) from a selection of oocytes at each stage of development. Singular measurement was constrained to those oocytes that were not distorted by the fixing process which was especially evident among the more developed stages. Where distortion occurred, 3 diameter measurements were made to provide an acceptable average diameter. Selection was also limited to oocytes displaying a nucleus with the assumption that it was situated towards an axis of the oocyte. Post ovulatory follicles could not be measured due to their convoluted shape.

### Plankton tows

Larval billfish sampling took place over several days in October and November 1988 and 1989 in the vicinity of the recreational fishing fleet supplying adult specimens for the reproductive component of this study. A conical net of 1 mm mesh was hung from a 1 m diameter frame and towed clear of the wake from a stern quarter of a 6 meter boat at a nominal 2 knots for 30 minutes (∼1800 m). The frame was buoyed to position the net immediately below the sea surface.

The topography of No. 10 Ribbon Reef, a particularly long barrier reef of 30 kms, includes an embayment approximately 2.5 kms south of the northern extent and referred to by gamefishers as First Corner (Fig. 1). A further 3.5 kms south, the reef takes another turn at Second Corner (Fig. 1). The prevailing southeasterly winds drive surface water north, against the set of the East Australian Current (EAC), and may form eddies in these embayments. The net was deployed along the extent of these embayments and also in a southerly facing embayment on Yonge Reef 8 kms north (Fig. 1).

The contents of the cod end from each tow were preserved in 70% ethanol. Fish larvae were separated and retained from each sample and billfish larvae were identified by Jeff Leis of the Australian Museum based on head profile and pigmentation as applied in Leis et al. [9]. Six larvae identified as black marlin based on these visual characters were submitted to DNA analysis (400 bases of the mitochondrial DNA control region compared to known black marlin sequences).

Measurement software and calibrated imagery were used to provide standard lengths for each larva to 0.1 mm following rehydration. This study did not have the scope to age larvae but, it was deemed useful to have some estimate of age in order to comment on the likelihood of larvae hatching at some distance from the capture location. Aging of larval black marlin has not been undertaken but age-length relationships for larval blue marlin were available. Prince et al. [15] aged Atlantic blue marlin from otolith microstructure and characterized growth with the Gompertz equation. Their smallest larval specimen was 6.22 mm and Serafy et al. [16] considered their calculated hatch size of 0.51 mm as an underestimate and chose 2.5 mm based on the smallest larva recorded by Leis et al. [9] and applied an exponential growth model to larvae <6.22 mm. The smallest istiophorid larva recorded in this study was 1.7 mm and this value was used as size at hatching. An instantaneous growth rate of 0.0976 [16] enabled calculated age estimates to be assigned to each larva. Sponaugle et al. [17], building on Serafy et al.'s collections, but dissecting out and enumerating all otoliths, determined various instantaneous growth rates by location, year and larval age. One of the higher rates (0.1280) was used to bracket the ages of larvae in this study.

### Popup Tagging

Popup satellite archival transmitting (PSAT) tags were deployed on black marlin from recreational fishing boats fishing the GBR between Cairns and the Ribbon Reefs. Marlin were captured via rod-and-reel by trolling natural baits along the reef. Once a marlin was hooked and subsequently controlled alongside the vessel, the condition of the fish was assessed; condition factors that would eliminate an individual fish from the tagging study included visible bleeding from the mouth or gills, hook in the gut or throat, or exhaustion that prevented the fish from maintaining upright while swimming. Fish that were determined to be in good health were tagged using a hand-held tagging pole to insert a nylon dart into the dorsal musculature at the base of the tallest portion of the first dorsal fin. Tags were rigged with an umbrella-style dart (described in [18]) and 136 kg test Sufix Superior monofilament (Yao I Fabric). The dart was inserted to a depth of 9.5 cm such that the nylon leader exited the body of the fish at a 45° angle towards the posterior of the fish.

PSAT tags from both Wildlife Computers (WC-Redmond WA, USA) and Microwave Telemetry (MT-Columbia MD, USA) were used for this study. WC PSAT tag models included PAT0, PAT1, PAT2, PAT3, PAT5 and PAT6 and the original MT PTT-100 model. PSAT tags are intended to remain attached to the fish recording temperature, depth and light data until a preprogrammed date and time when an electrolytic release mechanism activates and causes the tag to detachment from the fish. Once released, the tag floats to the surface and transmits data to the Argos satellite array. WC data are transmitted in a summarized histogram form while MT data are transmitted as raw hourly values for the measured parameters.

The dispersal pattern of each WC tagged fish was estimated with an extended version of the TrackIt model [19]. This model estimates two positions per day using raw light time-series data and is independent of the tag manufacturer's light-based positional estimates. MT PSATs do not provide raw light data so the TrackIt model cannot be employed and we relied upon the geolocation solutions from MT.

Movement patterns were plotted on both a local and ocean-basin scale. For local movement analyses the GBR Environment (GBRE) was defined as the area encompassing the GBR, Queensland Plateau, Marion Plateau, Queensland and Townsville Troughs (Fig. 1). This was done for two reasons - light based geolocation estimates were not precise enough to discriminate between fish locations adjacent to or 200 km seaward of the reef and, fishery data indicated that the GBRE experiences higher catch per unit effort during the months of October through December [13].

Sea surface temperature (SST) records from the PSAT tags were used to determine the mean temperature experienced by black marlin within the GBRE. Daily SST values were obtained for each tagged fish remaining in the GBRE by accessing the PSAT transmitted temperature/depth data.

### Conventional Tagging

Industry and Investment New South Wales (I&I NSW), Australia, owns and maintains a conventional tagging database produced by the NSW Game Fish Tagging Program (GFTP). To gather migration data for select game fish species the GFTP has been distributing conventional tags to recreational fishers since 1973. A stainless steel anchor dart is used on larger species such as billfish and sharks [20]. Black marlin tagging data from the east coast of Australia were obtained from I&I NSW to compliment the PSAT tag results. When compiling data for use in this study, tag recaptures that were missing location data or estimated size at both release and recapture were excluded. When analyzing movement of mature fish, recaptured fish that were a minimum of 100 kg upon release or recapture were included in the analyses. This was done to exclude all immature fish, based on the fact that females mature around 100 kg. Since males mature at a much smaller size (40 kg), certainly some mature males were unavoidably excluded from the analyses of mature fish movements.

## Results

### Staging of Oocytes

**Figure 2:**
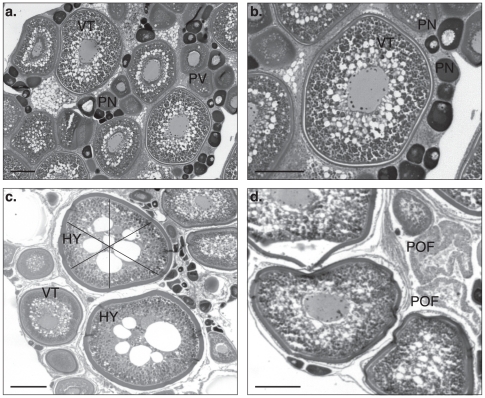
Histology of Spawning Black Marlin Ovary. A) Perinucleolar oocytes (PN), previtellogenic oocytes (PV) and vitellogenic oocytes (VT) displaying incremental development within these stages. B) Vitellogenic oocyte displaying a prominent nucleus with numerous peripheral nucleoli surrounded by yolk granules. C) early stage hydrated oocytes (HY) with large yolk globules and the 3 axes used to measure distorted oocytes. D) Post ovulatory follicles (POF) alongside advanced vitellogenic oocytes. Scale bar 200 µm.

**Figure 3:**
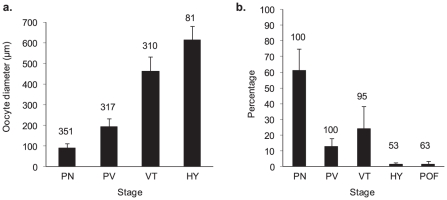
Size and relative frequency of black marlin oocyte stages. A - Mean sizes of oocyte development stages (+ 1SD) pooled from 19 black marlin taken from the Ribbon Reefs. The numbers above the bars indicate the number of oocytes measured. B – The mean proportional distribution of the various oocyte development stages in a fish (+ 1SD) compiled from 9312 egg counts. The numbers above the bars indicate the percentage of fish with each of the development stages. Stage designations are translated in Figure 2.

Four development stages were identified in the ovaries of 19 black marlin from the Ribbon Reefs: 1) perinucleolar (PN), 2) previtellogenic (PV), 3) vitellogenic (VT) and 4) hydrated (HY) oocytes (Fig. 2). Post ovulatory follicles (POF), indicative of recent spawning, were also recorded. Perinucleolar oocytes averaged 90 (21 SD) µm and ranged between 40 and 170 µm (Fig. 3A). The cytoplasm of these oocytes was heavily stained, with a relatively large nucleus and several nucleoli typically located peripherally in the nucleus. Previtellogenic oocytes averaged 192 (42 SD, range 90–130) µm with lipid droplets peripherally present and a membranous zona radiata. Vitellogenic oocytes averaged 460 (70 SD, 300–660) µm and were characterized by optically dense yolk globules and translucent lipid globules; the latter distributed towards the inner region of the cytoplasm. Only early stage hydration of oocytes was identified with large lipid droplets and a nucleus that had migrated from the centre of the oocyte. These oocytes averaged 615 (70 SD, 470–820) µm. Post ovulatory follicles were identified in 12 of the 19 fish examined. All but one of the 19 fish from the Ribbon Reefs contained vitellogenic oocytes and this 373 kg fish was taken early in the season (mid September) and was in a resting phase. It was evident from the distribution and occurrence of the oocyte development stages that the remaining fish were mature females and approximately half were very close to spawning or had done so recently (Fig. 3B).

All female fish from the nearshore coastal waters, inside the GBR, exhibited early stage perinucleolar oocytes and no evidence of recent or imminent spawning.

### Gonad weights

**Figure 4:**
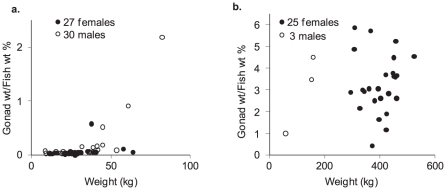
Gonad weights as a proportion of fish weight for black marlin taken from the inshore coastal waters (Fin – females, Min - males) and the Ribbon Reefs (Foff – females, Moff - males).

Testes of black marlin taken from the nearshore coastal waters were almost exclusively below 1% of whole fish weight and averaged 0.18% (0.08 SE). The exception was an 82.5 kg fish taken off Dunk Island that had relatively large testes of 1.8 kg (2.18%) (Fig. 4A). As the majority of males came from the Cape Bowling Green billfish grounds (25/30), no assessment of regional difference in relative testes weights were made. Ovaries of fish from the coastal waters averaged 0.06 (0.02 SE) % of fish weight. Similar to males, the nearshore sample of females was heavily weighted to Cape Bowling Green (19/26) and the only notable regional difference was a 38 kg fish from Dunk Island recording the heaviest absolute and relative ovary weight for a nearshore sample (0.22 kg and 0.58%).

The testes of 3 fish from the recreational heavy tackle fishery along the Ribbon Reefs averaged 2.98% (1.27 SE) of fish weight (Fig. 4B). The 2 larger fish (154.0 and 158.2 kg), more typical of males taken in this area, had a relative testes weights of 3.47% and 4.49%, respectively. Ovaries of fish from the Ribbon Reefs averaged 3.26% (0.27 SE) of fish weight with a maximum of 5.84% for one of the smaller females (309 kg). Ovary weights averaged 13.1 (1.18 SE) kg with the heaviest at 23.8 kg for the largest fish at 525 kg.

### Larvae

Thirty-one plankton tows produced 55 billfish larvae, accounting for 7.8% (5.6 SD) of fish larvae trapped by the net. Billfish larvae were recovered from 71% of tows and captured at the rate of 1.25/1000 m^3^ (1.23/m^3^ SD, range 0–4.95/m^3)^. The abundance of istiophorid larvae varied with the abundance of other fish larvae (r^2^ = 0.75) but no relationship with tide, time of day or moon phase was detected.

**Figure 5:**
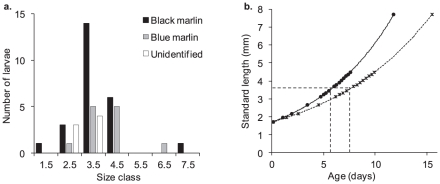
Age and growth of istiophorid larvae captured by towed plankton nets off the Ribbon Reefs. A - Size classes (midpoints) for istiophorid larvae captured by towed plankton nets off the Ribbon Reefs. B - Calculated ages for the larvae based on instantaneous growth rates for blue marlin of 0.0976 (lower curve after [16]) and 0.1280 (upper curve after [17]). Dotted lines indicate the median size and corresponding calculated ages.

The 55 billfish larvae included five swordfish, *Xiphias gladius*, between 5.5 and 21.8 (median 8.1) mm SL, and 50 istiophorid larvae between 1.7 and 7.7 mm SL (median 3.6 mm) with a strong modal class of 3–4 mm (Fig. 5A). Based on Ueyanagi's visual characters [8], [9], istiophorid larvae were assigned to black marlin (27), blue marlin (16) and unresolved (7). Six of the presumed black marlin were genetically tested and returned 3 black marlin, 2 blue marlin and 1 undetermined, thereby invalidating the visual identification method but validating the presence of black marlin larvae.

Application of growth rates borrowed from blue marlin gave istiophorid ages ranging from 0.1 to 15.5 days (median 7.6 days) and 0.1 to 11.8 days (median 5.8 days) at instantaneous growth rates of 0.0976 and 0.1280, respectively (Fig. 5B). The oldest swordfish larva (calculated after [21]) was 15.9 days which was little different to the maximum calculated age among the istiophorid larvae.

### PSAT Tagging

Sixty-seven PSAT tags were deployed on black marlin off the GBR (Fig. 1) between 2002 and 2009. Tags were placed in September (6), October (39), November (19) and December (3). Weights were visually estimated for 64 of the tagged marlin and ranged between 48–455 kg. The time at liberty for the PSAT tags ranged from 0–211 days. Forty-two data sets remained (tracks from 8–180 days) after excluding tags that were shed within the first week of deployment, did not transmit, transmitted poorly or were attached to fish that perished. Although programmed deployment lengths were relatively short (2–6 months), only 8 tags remained attached for the intended duration.

**Figure 6:**
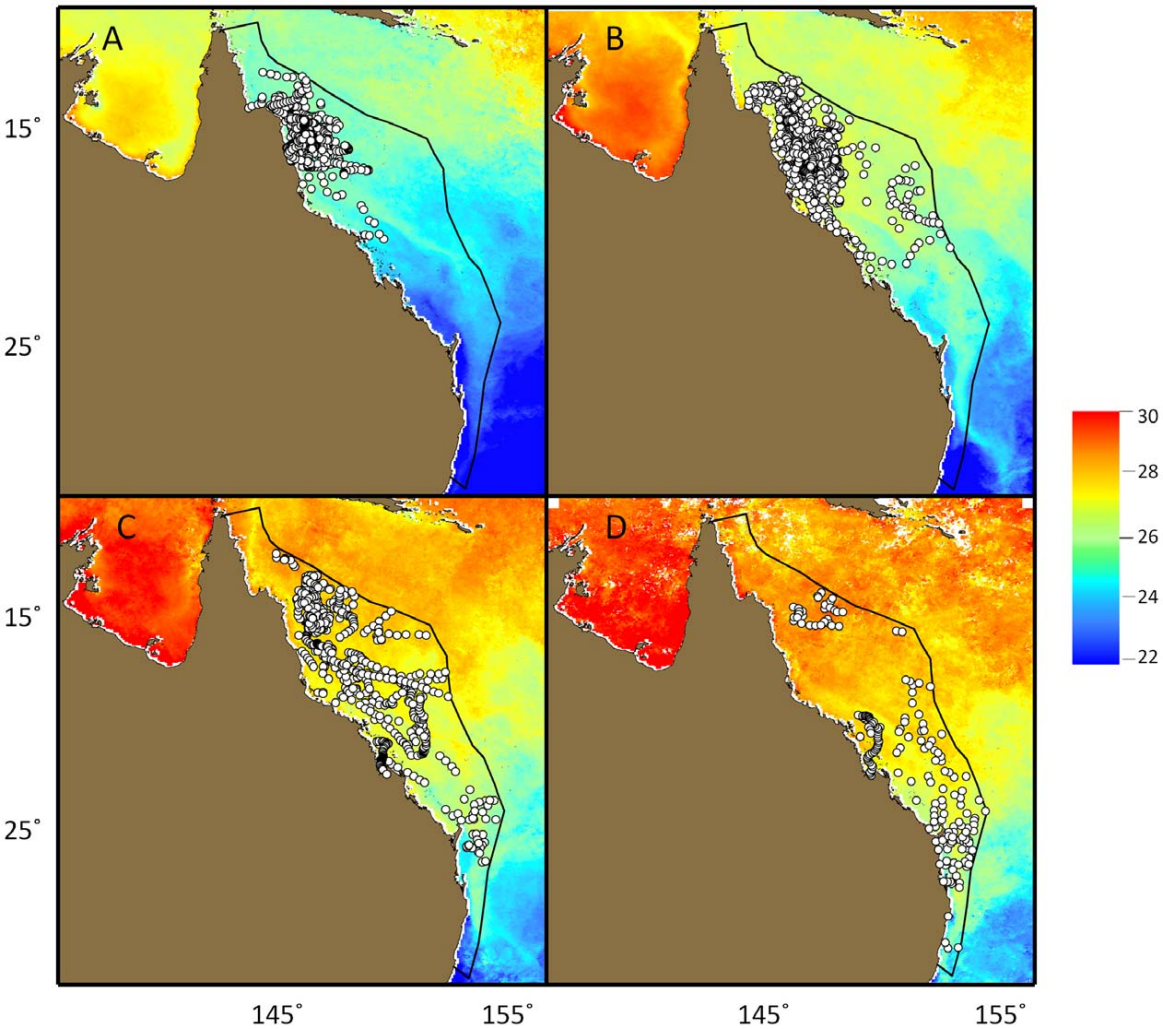
Movement of PSAT tagged black marlin within the GBRE, demonstrating an affinity for SST of approximately 26–27°C. A) October-November (n = 26 tagged fish); B) Dec-Jan (n = 27); C) February-March (n = 15); and D) April-May (n = 4). Sea Surface Representative monthly SST data from 2004–2005 MODIS (Moderate Resolution Imaging Spectroradiometer).

During October and November, marlin remained within the northern half of the Great Barrier Reef Environment (GBRE), but then dispersed southward as 26–27°C sea surface temperature (SST) spread south (Fig. 6). Mean SST experienced by PSAT tagged fish within the GBRE was 25.6°C in Oct. (n = 455 (discrete daily readings); 0.6 SD), 26.4°C in Nov. (n = 520; 0.5 SD) and 27.4 in Dec. (n = 345; 0.8 SD). The number of fish tracked within this area fell rapidly after November and only four fish remained after December (tags 6723, 05A0230, 05P0560, 05A0328). Continuous residencies within the GBRE ranged from 7–82 days (24 SD). One marlin (tag 05A0230) twice left and returned to the GBRE before the tag popped up near Ballina, New South Wales, Australia (29.0°S×153.5°E) 180 days after tagging. No tagged fish were tracked within the GBRE past April.

**Figure 7:**
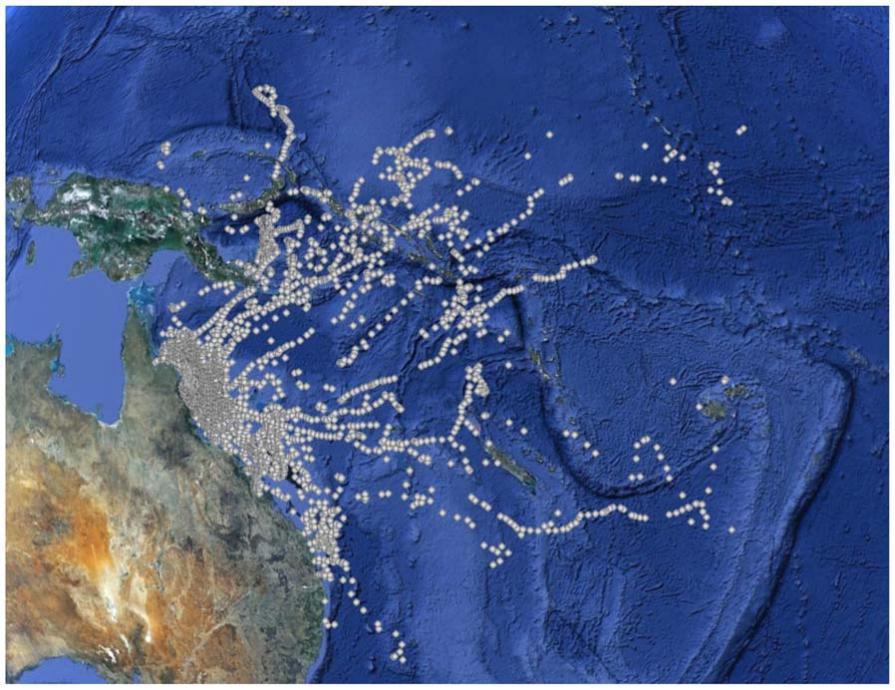
Catchment area of the GBR black marlin spawning aggregation, as determined by popup satellite tagging. Data SIO, NOAA, U.S. Navy, NGA, GEBCO; Image ©2011 TerraMetrics; Data © 2011 MIRC/JHA; © 2011 Cnes/Spot IMage.

Very few marlin left the GBRE during the months of October and November whereas in December and January all but one fish dispersed across the Coral Sea between a bearing of 350°T to 150°T (Fig. 7). More black marlin dispersed north through east (north of Vanuatu, n = 18) than east through south (south of Vanuatu, n = 8). Tagged fish dispersed to regions that included Papua New Guinea, Solomon Islands, Fiji, Vanuatu and the Gilbert Islands (Fig. 7). Only one fish remained near the Australian continent after January (tag 05A0230), briefly straying away from the continental shelf to the vicinity of Lord Howe Island before returning and shedding its tag in April. No marlin were tracked long enough to document return movement towards the GBRE for the subsequent spawning season.

**Figure 8:**
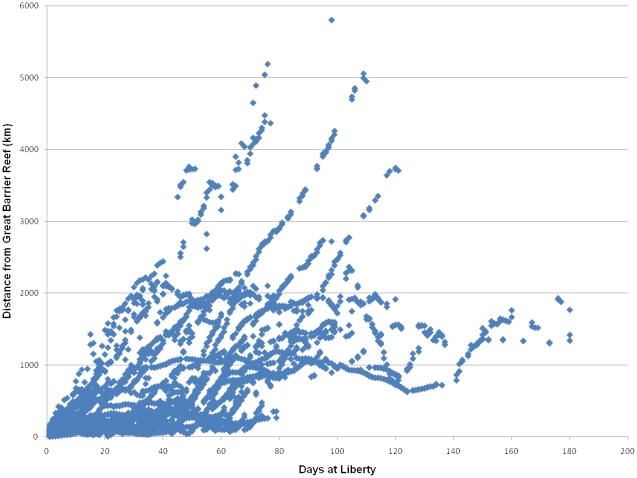
Range (km) of Black Marlin Dispersal from the GBR Spawning Aggregation.

Straight line track distances ranged from 822–5780 km (mean 2146 km, SD 1226 km) and path distances ranged between 2261–13431 km (mean 5016 km , SD 2158 km). Although a few marlin dispersed more than 2000 km from the GBR, calculation of the daily straight line dispersal distance revealed that most tagged marlin remained within 1000–2000 km of the GBR (Fig. 8). Average distances covered per day ranged from 21–137 km.

### Conventional Tagging

**Figure 9:**
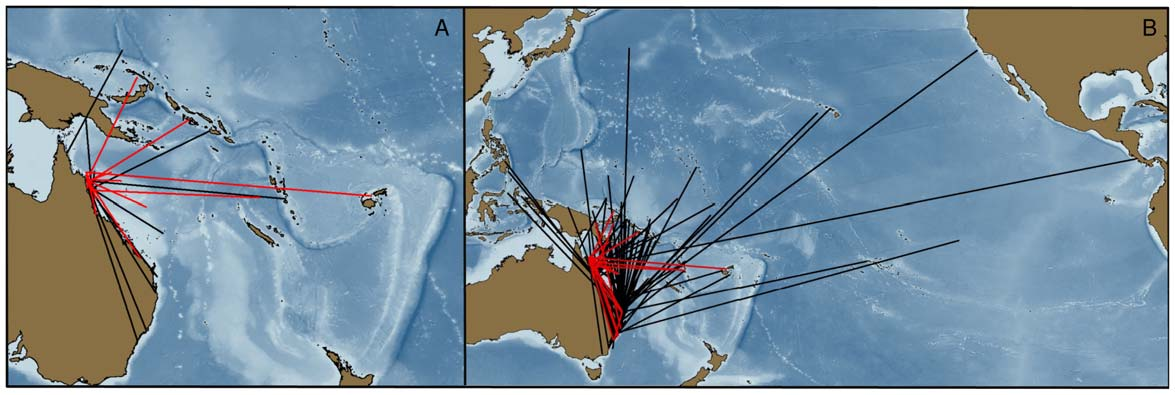
Conventional Tagging Results for Black Marlin Tagged off Eastern Australia. Recapture locations for black marlin tagged on the GBR (A) closely agree with popup tagging results; results of black marlin tagged throughout Australia (B) show some ocean basin crossings. Red tracks are those fish known to be mature (100+ kg).

Between April 1974 and November 2011 48297 black marlin were tagged with conventional tags and released off the east coast of Australia; 340 (0.7%) were subsequently recaptured. Thirty adult black marlin (100+ kg) tagged off the GBR were recaptured, demonstrating a similar dispersal pattern as the PSAT tags from this study (Fig. 9A). All mature fish (100+ kg) recaptured during the spawning season (n = 17; Oct-Nov.) were recaptured in the vicinity of the GBR (between 11and 1091 days at liberty). A wider range of dispersal was observed when recaptures from black marlin tagged throughout Australia were plotted (n = 337, Fig. 9B) with a few fish reaching the vicinity of Hawaii and the eastern Pacific.

## Discussion

### Evidence of Spawning

Our findings, which include the presence of hydrated eggs, post-ovulatory follicles and black marlin larvae, offer the first validation of a black marlin spawning aggregation. This spawning aggregation forms adjacent to the GBR during the months of October and November. Recreational fishing catch rates for black marlin are highest from Cairns north to the Ribbon Reefs, suggesting that this is the focus of spawning activity. Estimated ages for the istiophorid larvae collected for this study ranged from less than one day to about two weeks; a time frame that excludes the transport of larvae to the GBR from a distant spawning ground. However, incidental longline catches of black marlin 50–100 km offshore of the GBR during the spawning season indicate spawning could be occurring over a wide area. No ichthyoplankton sampling has occurred in the near vicinity of the reefs and islands dispersed throughout the Queensland Plateau during the black marlin spawning season, but such work could add valuable data on the spatial extent of black marlin spawning.

Prior to this study, no direct evidence of black marlin spawning had been collected within the geographic range of this species. With the exception of Leis et al. [9], very few marlin larvae had been obtained from the tropical western north Pacific, tropical Indian Ocean, or the Coral Sea [22]. Larvae that had been assigned to black marlin ([9], [23] and this study) were done via the process of elimination (exact method never published) and the identification had never been validated via other means. Our attempt to validate these visual characters via mitochondrial DNA testing resulted in the identification of both black and blue marlin in our presumed black marlin sample, clearly invalidating the previously published method (e.g., [8], [9], [16]).

### Larval and Juvenile Habitat

The I&I NSW data for released black marlin show a year round presence of small black marlin along the east coast of Australia with the GBR lagoon being an important seasonal site for these juveniles. The presence of ideal black marlin nursery habitat within the GBR lagoon may be the selective pressure that has led to the formation of a spawning aggregation adjacent to the GBR. Larvae that hatch seaward of the outer barrier reefs may eventually advect into the GBR lagoon. One indicator of water movement from outside the reef to the lagoon is the surprising number of PSAT tags that were shed outside of the GBR but subsequently transported into the lagoon, eventually washing ashore where they were recovered by beachgoers. Modeling of the flow of water in and out of the passages between the Ribbon Reefs also supports the notion that the larvae hatching adjacent to the reef can take advantage of tidal jets infiltrating the reef matrix and be transported into the lagoon and retained [24]–[26]. Although the presence of juvenile black marlin within the GBR lagoon is well documented, the one attempt to collect larvae in the lagoon found them to be scarce [9]. Black marlin larvae may occur in patches within the large GBR lagoon, and older larvae may be capable of avoiding capture, making sampling difficult. More sampling must occur before conclusions can be drawn regarding the abundance of black marlin larvae within the lagoon.

Analysis of recreational data from the annual Lizard Island Game Fish Club's tournament between 1991 and 1999 identified a peak in strike rates when the water was approaching 26°C [27]. These authors also related a peak in the Japanese longline fleet's return on effort in this region with the SST at 26°C. Similarly, the PSAT tagged fish moved southward in concurrence with the 26–27°C isotherm. A positive correlation between istiophorid and general fish larvae abundance, inferred from plankton tows, suggests that these pelagic and highly migratory species have a similar SST preference for spawning. Given a marlin's rapid ontogenetic shift to piscivory [28], the simultaneous spawning of marlin and reef species may not be coincidental. Also of interest is the concurrent presence of swordfish and blue marlin larvae which evidently encounter favorable conditions for survival and growth.

### Catchment Area and Black Marlin Movement Patterns

Marlin exhibit a high rate of PSAT tag shedding and therefore marlin studies that use this technology have very low sample sizes for tracks that exceed 6 months (e.g., [29]). Although premature tag shedding was an issue here, our tracking data provide a good indication of dispersal from the spawning aggregation while outlining the catchment area of the GBR spawning stock. Of note is the fact that no black marlin moved from the GBR to the South China Sea, Indian Ocean or eastern Pacific. Furthermore, no conventionally tagged black marlin have moved from the west coast of Australia (Indian Ocean) to the Indo Pacific, but there has been movement from the west coast of Australia to India (P. Bolton pers. comm.). Although mitochondrial DNA, microsatellite DNA and anonymous single copy nuclear DNA studies have failed to find any genetic stock structure between these important regions of black marlin abundance [30], [31], the GBR stock appears discrete from a practical fishery perspective.

The catchment area for the GBR stock is comprised of Papua New Guinea, the Solomon Islands, Micronesia, New Caledonia, Kiribati, Vanuatu, Fiji, Tuvalu and Nauru. A larger sample size and longer tracks would likely identify other adjacent regions, but the majority of this stock probably resides within our described catchment area during the non-spawning season. This hypothesis is supported by the lack of a clear relationship between the dispersal distance and days-at-liberty; although a few individuals continuously dispersed away from the GBR, most traveled 1000–2000 km before remaining at a relatively constant distance from the site of tagging (Fig. 8). Similarly, the majority of conventional tag returns from the I&I NSW program have come from the margins of the Coral Sea with a few long range movements of black marlin from Australia to Hawaii, the Philippines, French Polynesia and the eastern Pacific ( Fig. 9). These long range movements are rare and may be influenced by El Nino events [13]. The observed low levels of long range movements explain the global lack of genetic population structure.

Conventional tag data revealed evidence of spawning site philopatry. Adult black marlin tagged on the GBR during the spawning season were recaptured during subsequent spawning seasons in the same region. Whether spawning site fidelity occurs at other black marlin spawning grounds is not known. The identification of other regional black marlin spawning sites and subsequent PSAT tagging could further clarify our understanding of this species and its interaction with incidental and directed fisheries. Taiwan has previously been identified as a spawning region [6], but port sampling has not produced any adult fish in spawning condition (Chi-Lu Sun pers. comm.) and fish targeted at nearby Yanaguni Island, Japan, have been described as a feeding aggregation [32]. Hainan Island may be a spawning site for the South China Sea [10]–[12]. Black marlin are also abundant in the Indian Ocean, Sulu Sea, Celebes Sea and Philippine Sea but spawning sites are not presently known. No black marlin in spawning condition have ever been observed in the eastern Pacific [33].

The GBR is an important region for the reproduction and early life history of the black marlin. Protective measures that have been put in place to curtail the take of this species during the spawning season are likely important for the sustainability of this Coral Sea stock. The impact of fisheries that intercept black marlin migrating to/from the spawning grounds should be monitored to insure the health of this stock. The results presented here indicate the importance of tagging studies in addition to analyses of molecular markers, since genetic results alone may not provide results at a level meaningful to fisheries management. Several basic life history parameters are still not well defined for this species (e.g., minimum size of maturity, fecundity, spawning seasons/sites outside of GBRE) and future work should attempt to fill these gaps.
